# Long noncoding RNA PR11-387H17.6 as a potential novel diagnostic biomarker of atherosclerotic renal artery stenosis

**DOI:** 10.1080/0886022X.2021.1956537

**Published:** 2021-08-09

**Authors:** Wenxia Fu, Xiaoxiang Tian, Liwen Liu, Xiaolin Zhang, Xiaozeng Wang

**Affiliations:** a Department of Cardiac Function, Shanghai Chest Hospital, Shanghai Jiao Tong University, Shanghai, China; b Department of Cardiology and Institute of Cardiovascular Research, General Hospital of Northern Theater Command, Shenyang, China

**Keywords:** Long noncoding RNAs, atherosclerotic renal artery stenosis, biomarker, risk factor

## Abstract

**Background:**

Atherosclerotic renal artery stenosis (ARAS) is frequently related to ischemic nephropathy, secondary hypertension, and end-stage renal failure. Thus, this study aimed to explore whether certain circulating long noncoding RNAs (lncRNAs) may be used as potential specific ARAS biomarkers.

**Methods:**

In the present study, a microarray analysis was performed to screen for lncRNAs in renal artery tissue from four ARAS patients and four non-ARAS individuals. To identify specific lncRNAs as candidate potential biomarkers of ARAS, we used the following criteria: the fold change was set to >3.0 (compared with non-ARAS tissues), and *p* value cutoff was set at .05. According to these criteria, six lncRNAs were identified from 1150 lncRNAs. After validation by quantitative PCR (qPCR), these lncRNAs were independently validated in blood from groups of 18 ARAS patients, 18 non-ARAS individuals, and 18 healthy volunteers, furthermore, the predictive value of lncRNA PR11-387H17.6 was further assessed using blood from groups of 99 ARAS patients, 49 non-ARAS individuals, and 50 healthy volunteers. A receiver operating characteristic (ROC) curve analysis was performed to assess the performance of these lncRNAs as biomarkers.

**Results:**

In the ROC analysis, the area under the curve (AUC) of PR11-387H17.6 was 0.733, with 52.5% sensitivity and 84.8% specificity in predicting the occurrence of ARAS. After considering the risk factors, the AUC of PR11-387H17.6 was 0.844, and the optimal sensitivity increased from 52.5% to 74.5%, although the specificity decreased from 84.8% to 81.9%. In the multivariable logistic analysis, PR11-387H17.6 was an independent predictor of major adverse events (OR: 3.039; 95% CI: 1.388–6.654; *p*= .006).

**Conclusions:**

PR11-387H17.6 is a potential diagnostic biomarker of ARAS. The lncRNA levels in blood cells are regulated in ARAS. Thus, further investigations of the role of lncRNAs in ARAS are warranted.

## Introduction

Renal artery stenosis (RAS) is generally defined as a reduction in the luminal diameter in one or both renal arteries [1]. Stenosis of the renal arteries is mainly caused by atherosclerotic lesions that may result in progressive renal artery occlusion [2]. The prevalence of atherosclerotic renal artery stenosis (ARAS) varies from 1 to 5% in unselected populations with hypertension and 15–40% in populations with other manifestations of atherosclerosis, such as peripheral vascular disease (PVD) and coronary artery disease (CAD) [3]. However, patients with ARAS often do not present any clinical signs or symptoms, and ARAS is frequently related to ischemic nephropathy, secondary hypertension, and end-stage renal failure [4]. Angioplasty with renal artery stenting is an effective treatment strategy for hemodynamically significant ARAS that restores and preserves renal function and controls blood pressure [5]. However, recent studies comparing renal endovascular revascularization to medical therapy have shown that stenting procedures performed in conjunction with medical therapy do not improve cardiovascular and renal outcomes [6]. Invasive screening for ARAS is very expensive, particularly when conducted simultaneously with another invasive diagnostic procedure, such as cardiac catheterization, and may affect treatment strategies. Currently, many patients do not visit a hospital for treatment until they experience symptoms, which increases the likelihood of complications. Therefore, the development of a new noninvasive method that may be used to diagnose ARAS is critical to enable effective evidence-based medical management and treatment.

Long noncoding RNAs (lncRNAs) constitute a novel class of molecules that range from 200 to over 10,000 nucleotides and lack the ability to code proteins [7]. The recognition of the roles of lncRNAs in human disease has provided a mechanistic understanding and could lead to novel diagnostic and therapeutic approaches [8]. LncRNAs often form secondary structures and are relatively stable, facilitating their detection as free nucleic acids in body fluids, such as blood [9]. Recently, thousands of lncRNAs have been found in different species. Accumulating evidence suggests that lncRNAs play crucial roles in controlling gene expression and other cellular processes during development and differentiation [10]. Several lncRNAs are also involved in the development of cardiovascular diseases [11,12], including heart failure [13,14], cardiac hypertrophy [15,16], cardiometabolic diseases [17], and myocardial infarction [18], and atherosclerosis severity [19,20]. Moreover, lncRNAs can be used as biomarkers of several cardiovascular diseases. For instance, the lncRNA LIPCAR is used to detect heart failure after myocardial infarction [13]. Circulating levels of certain lncRNAs, such as cyclin-dependent kinase inhibitor 2B antisense RNA 1 (ANRIL) and LincP21, are markedly increased in atherosclerosis, which may be important for its pathogenesis [19]. Such findings provide evidence regarding the potential roles of lncRNAs in the development and progression of atherosclerosis. In the present study, we investigated whether certain circulating lncRNAs can be altered in patients with ARAS, and may offer a potential component for the study of ARAS.

Thus, the aim of our study was to identify specific circulating lncRNAs that may serve as potential ARAS biomarkers. LncRNAs were screened by a microarray analysis and validated in CAD patients and healthy volunteers in different cohorts with or without ARAS.

## Materials and methods

### Study cohorts

This study used a single-center study designed to advance the diagnosis of ARAS at the General Hospital of Northern Theater Command. In total, 121 patients with ARAS, which was defined by the following:(1) aged 18–80 years; (2) one of RAS ≥50% and coronary artery lesions graded as ≥50% narrowing of luminal diameter, 71 patients without ARAS (<50% or no luminal narrowing) undergoing percutaneous coronary angiography and renal arteriography were enrolled in this study between 1 May 2015 and 31 September 2016. Simultaneously, arterial blood samples were collected from 68 apparently healthy volunteers (i.e., without apparent signs of cardiovascular disease, peripheral arterial disease, and cardiovascular risk factors). The initial ‘microarray cohort’ comprised renal artery tissue from a discovery group of four ARAS patients and four non-ARAS individuals. The association between the lncRNA expression and the risk of ARAS was analyzed in two separate cohorts. The training group comprised 18 ARAS patients, 18 non-ARAS individuals, and 18 healthy volunteers. To investigate the specificity of the association between PR11-387H17.6 and ARAS, a validation group comprising 99 ARAS patients, 49 non-ARAS individuals, and 50 healthy volunteers was also analyzed. The exclusion criteria for this study were as follows: (1) renal size <7.5 cm on the stenotic side; (2) age less than 18 or greater than 80 years; (3) eGFR <15 mL/min; (4) malignant tumors or other severe systemic diseases (such as renal failure or hepatic disease); (5) serious acute infection within 6 weeks before admission; (6) active chronic inflammatory disease; and (7) suspected drug or alcohol abuse. The diagnosis was based on the final diagnosis based on coronary and renal artery angiography at discharge according to the ACC/AHA classification [21]. The angiographic findings were interpreted independently by two blinded interventional cardiologists in a blinded manner. The study was conducted in accordance with the Declaration of Helsinki and was approved by the local ethics committee of the hospital (K-2014-29). Written informed consent for surgery was obtained from all patients or their families.

### Preparation and isolation of peripheral blood leukocyte samples

After angiography, peripheral blood samples (5 mL) were collected from the radial arteries of each patient in test tubes containing EDTA. The hematocytes were carefully collected, divided into aliquots and stored at −80 °C before use. The total RNA was extracted using TRIzol Reagent (Invitrogen Life Technologies, Carlsbad, CA) according to the manufacturer’s instructions. All steps were performed at 4 °C.

### RNA isolation and qPCR

The total RNA was extracted from the white blood cells and dissolved in 10 µL of DNase/RNase-free deionized water (TIANGEN, Beijing, China). The quantity and quality of the total RNA from the peripheral blood were determined using a NanoDrop instrument (Agilent, Santa Clara, CA), and the samples were used only if the ratio of the absorbance at 260 and 280 nm (A260/280) was between 1.5 and 1.9. RNA samples at concentrations of 1 μg were used for each reverse transcription reaction. Then, the RNA was used for the cDNA synthesis using a PrimeScript 1st Strand cDNA Synthesis Kit (TaKaRa, Dalian, China) according to the manufacturer’s instructions. Then, quantitative real-time PCR was conducted using EvaGreen 2X qPCR MasterMix (Applied Biological Materials Inc., Richmond, Canada) according to the manufacturer’s instructions. The 20 µL final reaction mixture contained 10 µL of EvaGreen 2X qPCR MasterMix, 0.6 µL of forward primer, 0.6 µL of reverse primer, 6.8 µL of nuclease-free H_2_O, and 2.0 µL of the synthesized cDNA. The reaction conditions were as follows: 95 °C for 10 min, followed by 35 cycles of 95 °C for 15 s and 60 °C for 60 s. The Ct value was defined as the cycle number at which the fluorescence exceeded the threshold. The levels of the lncRNAs in the peripheral blood were calculated using the CT (ΔCt) method because currently, no consensus exists regarding stable and suitable internal controls for lncRNAs in blood samples. The changes in gene expression were calculated using the equation 2^–ΔCT^ [22].

The relative expression levels of the lncRNAs in the peripheral blood leukocytes were normalized to the expression of the endogenous control gene glyceraldehyde-3-phosphate dehydrogenase (GAPDH) (Sangon Biotech, Shanghai, China) using the comparative CT (ΔCt) method. The lncRNA levels were log-transformed by considering the base 10 logarithm of the skewness of their distribution. A melt curve analysis was performed to confirm the specificity of the amplification and lack of primer dimers. The quantitative PCR (qPCR) primers for the lncRNAs are listed in Supplementary Table S1.

### Microarray and computational analysis

For the initial lncRNA screening, RNA was isolated from samples of renal artery tissue collected during a nephrectomy surgery from eight individuals (four ARAS patients and four non-ARAS individuals). Written informed consent for the surgery was obtained from the eight patients or their families. The total RNA from each sample was quantified using a NanoDrop ND-1000. The RNA was pre-amplified and was subjected to a LncPath™ Human Cardiovascular Disease Pathway LncRNA Array (Arraystar, Rockville, MD), which allowed for the simultaneous detection of 1150 lncRNAs and 1673 coding transcripts. After filtering the low-intensity lncRNAs, the lncRNAs in the eight samples were subjected to quantile normalization and supplementary data analysis. The quantile normalization and subsequent data processing were performed using the open-source R software package. Agilent Feature Extraction software (version 11.0.1.1) was utilized to analyze the acquired array images. Differentially expressed lncRNAs that statistical significantly differed between two groups were defined by Volcano Plot filtering. The differentially expressed lncRNAs between two samples were identified by fold change filtering. Hierarchical clustering was performed to show the distinguishable lncRNA expression patterns among the samples. To identify a potential biomarker candidate lncRNA, all lncRNAs were screened according to the following criteria: the fold change was set to >3.0 (compared with non-ARAS tissues), and *p* value cutoff was set at .05.

### Statistical analysis

The data are presented as the means ± SD or number (percentage) of patients. The continuous variables were compared using paired Student’s *t*-tests or non-parametric paired tests for abnormal distributions. The discrete variables were compared using chi-square or Fisher’s exact tests. A logistic regression analysis was performed to determine the demographic and clinical factors that predicted the endpoints. Multivariate logistic regression was utilized to calculate the odds ratios (ORs) and corresponding 95% confidence intervals. Univariate and multivariate logistic regression analyses were performed to analyze the independent risk factors among the ARAS patients. We generated receiver operating characteristic (ROC) curves to assess the diagnostic values of the six biomarkers. The area under the curve (AUC) was used as a measure of the diagnostic accuracy of the biomarkers. A *p* value<.05 was considered statistically significant. The statistical analyses were performed using SPSS Statistics 17.0 (IBM SPSS Inc., Chicago, IL).

## Results

### lncRNA expression profiles in tissue and peripheral blood cells from ARAS patients

**Figure 1.**
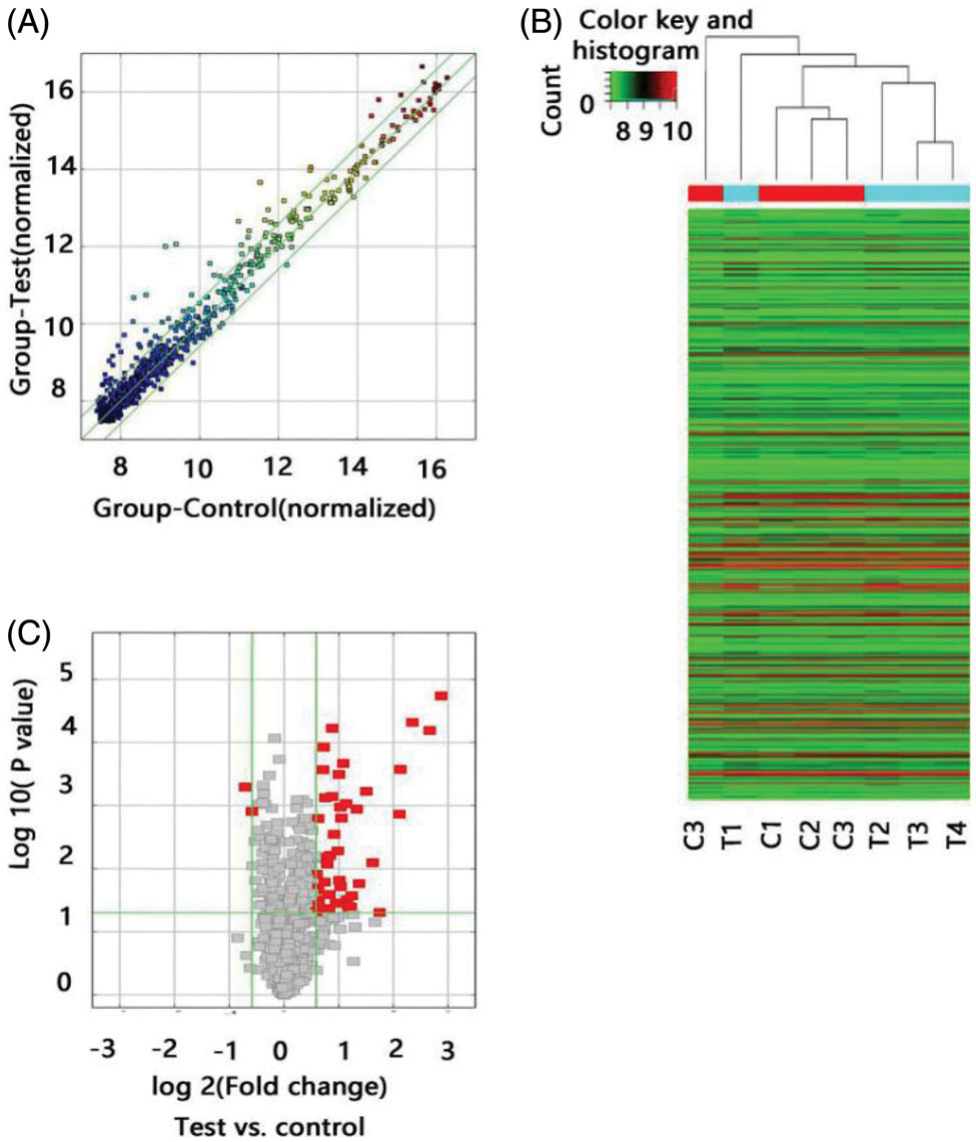
Differential expression of lncRNAs in ARAS patients and control individuals. (A) The scatter plots showed LncRNA expression in test samples versus normal samples. *X*-axis depicted data values of control samples; *Y*-axis depicted data values of test samples. Dots were located above the upper green line and below the under green line represent fold change ≥1.5, ‘Test’ indicates ARAS samples; ‘Normal,’ control samples. (B) Heat map of lncRNA expression from microarray analysis of combined renal artery tissue samples of patients with ARAS and control subjects (T, renal atherosclerosis tissue; C, normal renal artery tissue). Each row represented one lncRNA and each column represents a sample. The color scale shown at the top illustrated the relative expression level of a lncRNA; red represents high expression and green represented low expression. (C) The volcano plots showed thousands of lncRNAs were significantly different by using lncRNA expression thresholds of more than 1.5-fold change with *p*<.05. The red point in the plot represented the deferentially expressed Coding genes with statistical significance.

To determine whether specific lncRNAs were expressed in the ARAS patients, we profiled the tissue lncRNA expression in four ARAS patients and four non-ARAS individuals using the LncPath™ Human Cardiovascular Disease Pathway microarray. The lncRNA levels in the renal artery tissue significantly differed between the two groups as illustrated by the hierarchical clustering analysis (Figure 1). Of the 1150 lncRNAs detected in the microarray, 45 lncRNAs were differentially expressed in the ARAS patients with a fold change *>*1.5 and *p<* .05. To ensure that the potential lncRNA markers could be easily measured in the clinic, we selected biomarkers from the 43 upregulated lncRNAs using the following strategy: a fold change *>*3.0 and *p<* .05. Only the following six lncRNAs met these criteria: RP11-387H17.6, BC080653, RP1-32B1.4, RP5-1068H6.3, GHRLOS, and XLOC_009769.

### Independent validation of lncRNA expression

To validate the increased expression of RP11-387H17.6, BC080653, RP1-32B1.4, RP5-1068H6.3, GHRLOS, and XLOC_009769 in the peripheral blood cells from the ARAS patients, these lncRNAs were quantified in blood cell samples obtained from a training group comprising 18 ARAS patients, 18 non-ARAS patients, and 18 healthy volunteers. The clinical and demographic characteristics of the patients are shown in Table 1. The lncRNA primers used are listed in Supplementary Table S1.

**Table 1.** Characteristics of a training group population.

	Healthy volunteer (*n* = 18)	Non-ARAS (*n* = 18)	ARAS (*n* = 18)	*p* Value
Age, years	42.50 ± 8.50	58.00 ± 10.23*	64.56 ± 9.81*^,^^+^	<.001
Female, *n* (%)	7 (38.89)	5 (27.78)	6 (33.33)	.709
Physical data				
Body mass index, kg/m^2^	23.76 ± 2.49	26.89 ± 4.72*	25.49 ± 3.16	.046
Systolic blood pressure, mmHg	125.39 ± 13.81	135.56 ± 24.40	149.94 ± 13.10*^,^^+^	.002
Diastolic blood pressure, mmHg	74.39 ± 12.54	76.39 ± 13.68	77.44 ± 9.32	.741
Pulse pressure, mmHg	51.83 ± 5.77	59.44 ± 16.86	70.78 ± 12.31*^,^^+^	<.001
Smoking, *n* (%)	–	10 (55.56)	11 (61.11)	.735
Drinking, *n* (%)	–	2 (11.11)	4 (22.22)	.371
Laboratory data				
CK-MB, U/L	–	14.61 ± 5.20	17.28 ± 14.33	.466
WBC (×10^9^/L)	6.04 ± 1.33	7.64 ± 2.23*	7.98 ± 1.45*	.004
TC, mmol/L	4.44 ± 0.53	4.10 ± 0.88	4.91 ± 1.58^+^	.091
TG, mmol/L	0.90 ± 0.34	1.85 ± 0.60*	2.91 ± 1.96*^,^^+^	<.001
LDL, mmol/L	2.77 ± 0.41	2.34 ± 0.58	2.99 ± 1.21^+^	.058
HDL, mmol/L	2.33 ± 0.25	1.62 ± 0.30*	1.48 ± 0.33*	<.001
Creatinine, µmol/L	69.11 ± 13.40	76.05 ± 16.31	84.31 ± 19.16*	.456
eGFR, mL/min	127.70 ± 30.84	104.01 ± 26.15*	99.44 ± 5.34*	.001
NT-ProBNP, pg/mL	48.58	90.45	164.90*	.002
lncRP11-387H17.6, log change	0.02	0.02	0.15*	.001
Comorbid conditions, *n* (%)				
Prior MI	–	1 (5.56)	3 (16.67)	.596
Hypertension	–	8 (44.44)	14 (77.78)	.040
Diabetes mellitus	–	3 (16.67)	5 (27.78)	.688
Dyslipidemia	–	10 (55.6)	10 (55.6)	1.000
Stroke	–	2 (11.11)	5 (27.78)	.238
Peripheral artery disease	–	0 (0.00)	3 (16.67)	.070
Coronary artery disease vessels	–			
1 vessel	–	1 (5.56)	3 (16.67)	.596
2 vessel	–	5 (33.33)	7 (22.22)	.480
3 vessel	–	7 (38.89)	8 (44.44)	.735

ARAS: atherosclerotic renal artery stenosis; TC: total cholesterol; TG: triglyceride; LDL: low-density lipoprotein cholesterol; HDL: high-density lipoprotein cholesterol; eGFR: estimated glomerular filtration rate using the Chronic Kidney Disease Epidemiology Collaboration; NT-ProBNP: N-terminal pro-brain natriuretic peptide; MI: myocardial infarction.

Data are presented as means (±SD) or number (%).

* *p*<.05 vs. healthy controls.

+ *p*<.05 vs. non-ARAS.

**Figure 2.**
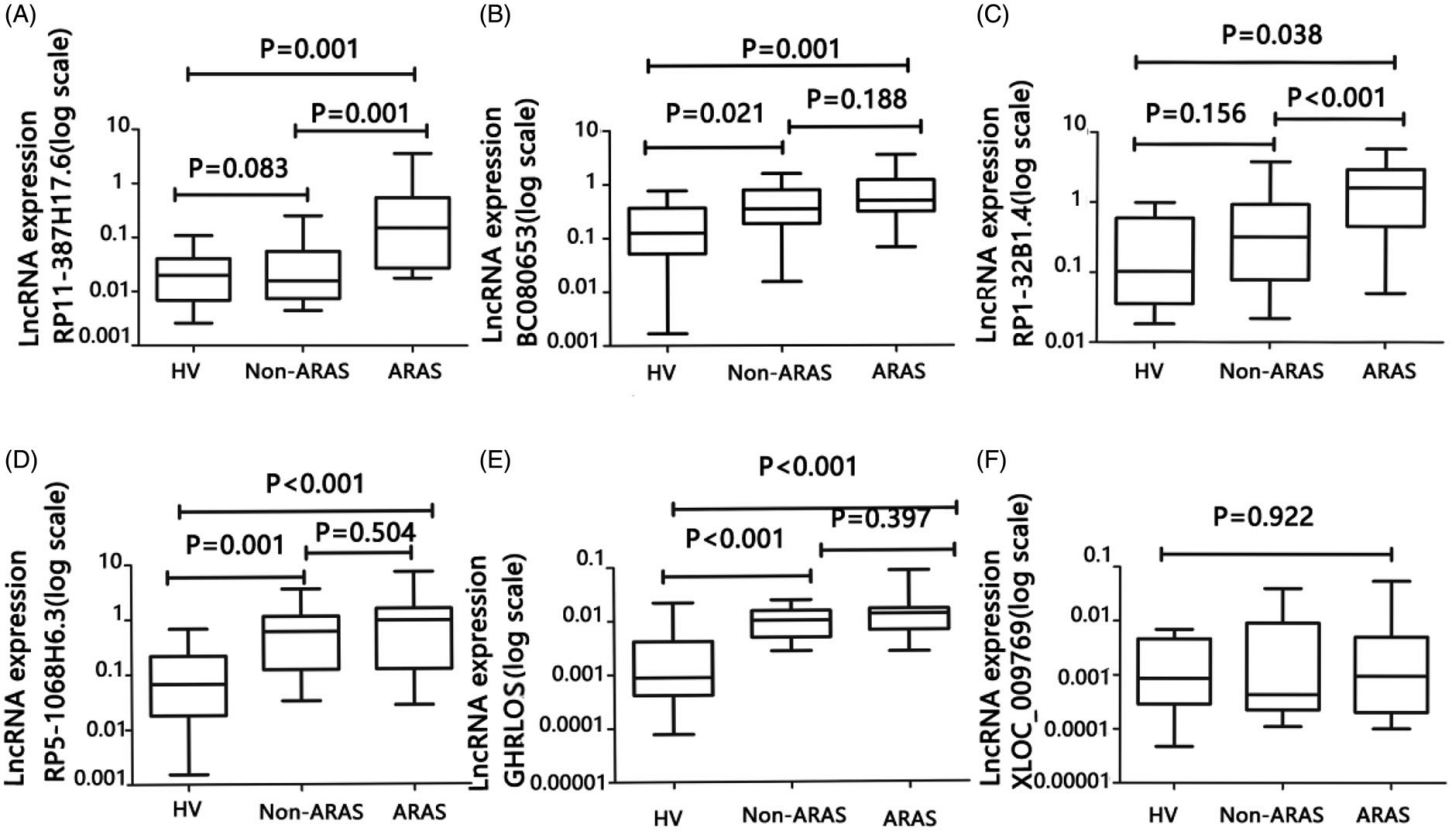
Expression levels of lncRNAs RP11-387H17.6, BC080653, RP1-32B1.4, RP5-1068H6.3, GHRLOS, and XLOC_009769 were assessed by quantitative polymerase chain reaction (qPCR) using GAPDH as a reference gene for normalization among patients with ARAS (*n* = 18), non-ARAS (*n* = 18), and HV(*n* = 18). (A–F) Expression levels of lncRNAs: (A) RP11-387H17.6, (B) BC080653, (C) RP1-32B1.4, (D) RP5-1068H6.32, (E) GHRLOS, and (F) XLOC_009769. ARAS: atherosclerotic renal artery stenosis; HV: healthy volunteers.

**Figure 3.**
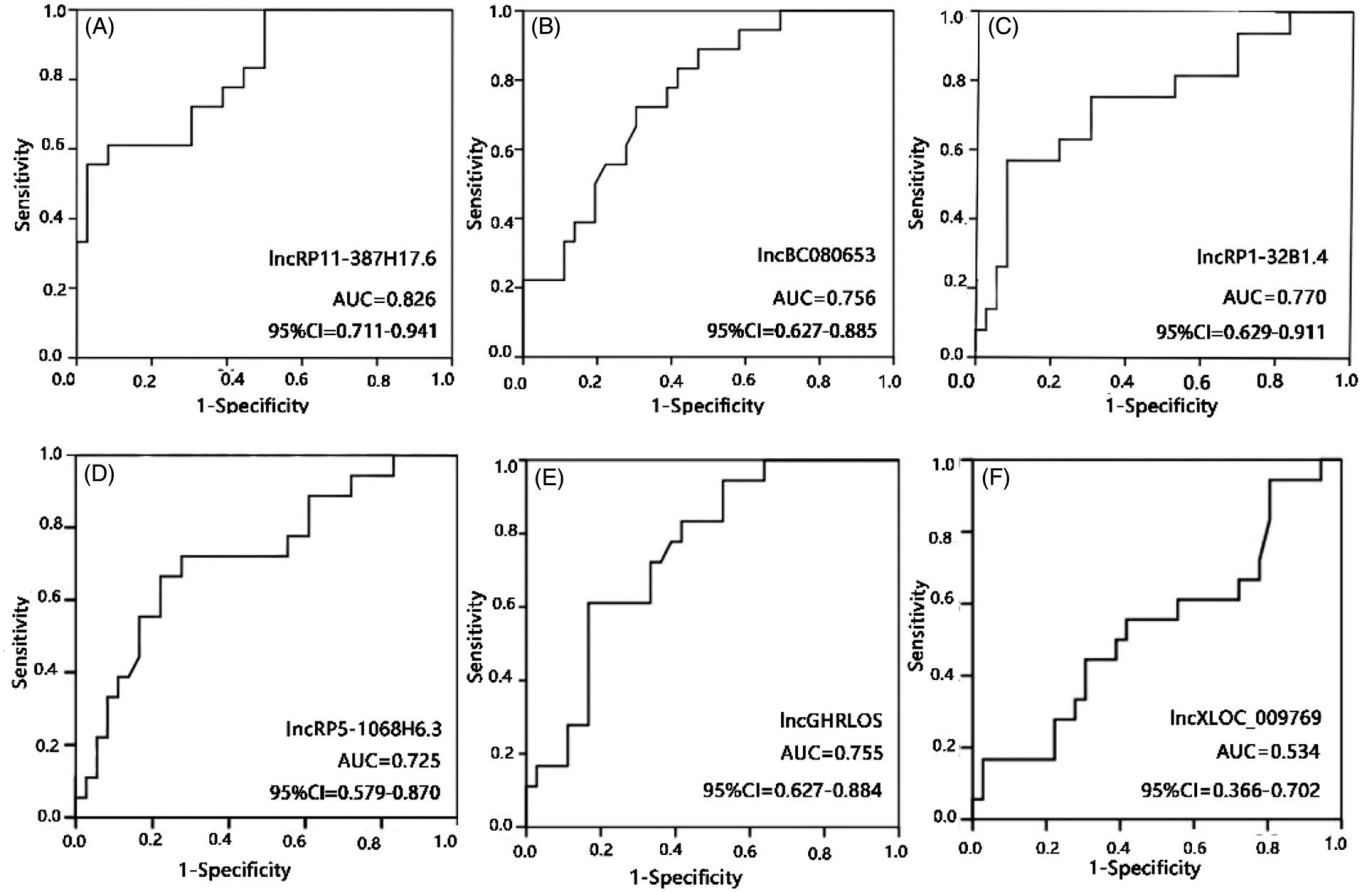
Receiver operating characteristic (ROC) curve analyses of LncRNAs RP11-387H17.6, BC080653, RP1-32B1.4, RP5-1068H6.3, GHRLOS, and XLOC_009769 for diagnosis of ARAS among patients with ARAS (*n* = 18), non-ARAS (*n* = 18), and HV (*n* = 18). AUC: area under the curve, ARAS: atherosclerotic renal artery stenosis. (A–F) Expression levels of lncRNAs: (A) RP11-387H17.6, (B) BC080653, (C) RP1-32B1.4, (D) RP5-1068H6.32, (E) GHRLOS, and (F) XLOC_009769. ARAS: atherosclerotic renal artery stenosis; HV: healthy volunteers.

To determine the relationship between these lncRNA levels and ARAS, an ROC analysis was performed. The AUCs were 0.826 for RP11-387H17.6, 0.756 for BC080653, 0.770 for RP1-32B1.4, 0.725 for RP5-1068H6.3, 0.755 for GHRLOS, and 0.534 for XLOC_009769 (Figures 2 and 3). These results indicate that RP11-387H17.6 may be a potential candidate biomarker for the diagnosis of ARAS.

### Additional clinical validation

**Figure 4.**
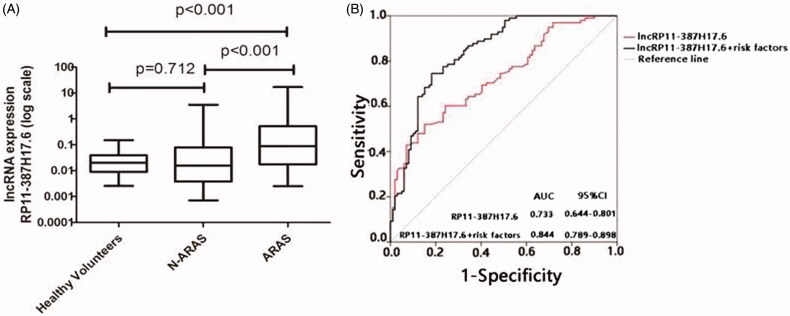
Expression levels of lncRP11-387H17.6 and ROC curve analyses of lncRP11-387H17.6 alone and lncRP11-387H17.6 combined with risk factors for the diagnosis of ARAS among patients with ARAS (*n* = 99), non-ARAS (*n* = 45), and HV (*n* = 50). (A) Expression levels of lncRNAs RP11-387H17.6 among patients with ARAS (*n* = 99), non-ARAS (*n* = 49), and HV (*n* = 50). GAPDH was used as the normalization control. (B) ROC curves showing the diagnostic performance of lncRP11-387H17.6 alone and lncRP11-387H17.6 combined with risk factors. ARAS: atherosclerotic renal artery stenosis; HV: healthy volunteers.

As the above analyses involved two different groups, we further assessed RP11-387H17.6 as a biomarker of ARAS in a large validation group (ARAS patients, *n* = 99; non-ARAS individuals, *n* = 49; and healthy volunteers, *n* = 50). The model and criteria used were the same as those used in the training group. The clinical and demographic characteristics of this population are summarized in Table 2. We performed an ROC analysis to evaluate the diagnostic ability of RP11-387H17.6. The diagnostic sensitivity and specificity for ARAS were 52.5% and 84.8%. The AUC of RP11-387H17.6 for ARAS was 0.733 (95% CI: 0.644–0.801), indicating that RP11-387H17.6 in peripheral blood cells may be a potential candidate marker of ARAS (Figure 4).

**Table 2.** Characteristics of a testing group population.

	Healthy volunteers (*n* = 50)	Non-ARAS (*n* = 49)	ARAS (*n* = 99)	*p* Value
Age, years	49.56 ± 11.97	60.67 ± 9.63*	64.85 ± 8.86*^,^^+^	<.001
Female, *n* (%)	18 (36.00)	11 (22.45)	40 (40.40)	.096
Physical data				
Body mass index, kg/m^2^	23.00 ± 3.39	26.91 ± 2.86*	25.46 ± 3.40*^,^^+^	<.001
Systolic blood pressure, mmHg	119.64 ± 12.03	138.00 ± 19.68*	146.23 ± 21.90*^,^^+^	.016
Diastolic blood pressure, mmHg	74.30 ± 9.23	77.43 ± 12.26	79.32 ± 12.58	.051
Pulse pressure, mmHg	45.34 ± 10.91	60.57 ± 16.53*	66.24 ± 20.82*	<.001
Smoking, *n* (%)	–	30 (61.22)	42 (42.42)	.031
Drinking, *n* (%)	–	17 (34.69)	19 (19.19)	.120
Laboratory data				
CK-MB, U/L	–	13.69 ± 8.69	14.81 ± 13.30	.594
WBC (×10^9^/L)	5.92 ± 1.61	7.08 ± 1.78*	7.31 ± 1.74*	<.001
TC, mmol/L	4.04 ± 0.58	3.95 ± 1.02	4.35 ± 1.09*^,^^+^	.038
TG, mmol/L	0.91 ± 0.36	2.09 ± 2.12*	1.94 ± 1.92*	.001
LDL, mmol/L	2.08 ± 0.58	2.29 ± 0.76	2.47 ± 1.00*	.032
HDL, mmol/L	3.08 ± 0.65	0.79 ± 0.16*	0.91 ± 0.39*	<.001
Creatinine, µmol/L	65.88 ± 13.35	77.45 ± 17.66*	77.85 ± 24.44*	.002
eGFR, mL/min	134.52 ± 43.46	98.95 ± 22.47*	94.02 ± 28.63*	<.001
NT-ProBNP, pg/mL	58.80	93.74*	188.60*^,^^+^	<.001
lncRP11-387H17.6, log change	0.02	0.02	0.09*^,^^+^	<.001
Comorbid conditions, *n* (%)				
Prior MI	–	17 (34.69)	19 (19.19)	.039
Hypertension	–	21 (42.86)	31 (31.31)	.084
Diabetes mellitus	–	31 (63.27)	76 (76.77)	.084
Dyslipidemia	–	33 (67.35)	48 (48.48)	.030
Stroke	–	8 (16.33)	27 (27.27)	.140
Peripheral artery disease	–	2 (4.08)	8 (8.16)	.354
Coronary artery disease vessels	–			
1 vessel	–	13 (26.53)	20 (20.20)	.439
2 vessel	–	17 (34.69)	35 (35.35)	.834
3 vessel	–	19 (38.78)	40 (40.40)	.737

Data are presented as means (±SD) or number (%).

ARAS: atherosclerotic renal artery stenosis; TC: total cholesterol; TG: triglyceride; LDL: low-density lipoprotein cholesterol; HDL: high-density lipoprotein cholesterol; eGFR: estimated glomerular filtration rate using the Chronic Kidney Disease Epidemiology Collaboration; NT-ProBNP: N-terminal pro-brain natriuretic peptide; MI: myocardial infarction.

* *p*<.05 vs. healthy controls.

+ *p*<.05 vs. non-ARAS.

### Univariate and multivariate logistic analyses of ARAS

We further assessed the relationship between the expression of lncRP11-387H17.6 and ARAS. The univariate analysis indicated that age, sex, systolic pressure, pulse pressure, cholesterol, LDL, and lncRP11-387H17.6 were significantly associated with ARAS among the patients (Table 3). Moreover, age, systolic pressure and lncRP11-387H17.6 expression were independent diagnostic factors of ARAS in the multivariate analysis (Table 4). Therefore, an additional ROC analysis was performed to examine the potential of lncRP11-387H17.6 as a biomarker of ARAS.

**Table 3.** Univariate analysis for lncRP11-387H17.6 in patients with ARAS.

	Univariate analysis	
Variable	OR (95% CI)	*p* Value
Sex	1.992 (1.015–3.908)	.045
Age, years	1.058 (1.022–1.095)	.001
SBP, mmHg	1.022 (1.006–1.037)	.005
Pulse pressure, mmHg	1.020 (1.003–1.038)	.021
Smoking	0.559 (0.304–1.028)	.061
Dyslipidemia	0.549 (0.296–1.017)	.057
TC, mmol/L	1.460 (1.088–1.960)	.012
LDL, mmol/L	1.714 (1.147–2.561)	.009
lncRP11-387H17.6, log change	3.249 (1.268–8.324)	.014
NT-ProBNP, pg/mL	1.000	.531
Prior MI	0.630 (0.309–1.285)	.204

ARAS: atherosclerotic renal artery stenosis; SBP: systolic blood pressure; TC: total cholesterol; LDL: low-density lipoprotein cholesterol; NT-ProBNP: N-terminal pro-brain natriuretic peptide; MI: myocardial infarction; OR: odds ratio; CI: confidence interval.

Dichotomous variables (yes = 1, no = 0): smoking, dyslipidemia, prior MI; categorical variables: sex (female = 1, male = 0).

**Table 4.** Multivariate analysis for lncRP11-387H17.6 in patients with ARAS.

	Multivariate analysis	
Variable	OR (95% CI)	*p* Value
Sex	1.209 (0.500–2.922)	.673
Age, years	1.051 (1.010–1.095)	.016
SBP, mmHg	1.028 (1.010–1.047)	.003
Smoking	0.697 (0.319–1.520)	.364
TC, mmol/L	1.373 (0.597–3.162)	.456
LDL, mmol/L	1.296 (0.432–3.886)	.644
lncRP11-387H17.6, log change	3.039 (1.388–6.654)	.006

ARAS: atherosclerotic renal artery stenosis; SBP: systolic blood pressure; TC: total cholesterol; LDL: low-density lipoprotein cholesterol; NT-ProBNP: N-terminal pro-brain natriuretic peptide; OR: odds ratio; CI: confidence interval.

Dichotomous variables (yes = 1, no = 0): smoking; categorical variables: sex (female = 1, male = 0).

### Combination with risk factors increased the diagnostic performance of lncRP11-387H17.6 as an ARAS signature

As noted above, we found an association between the lncRP11-387H17.6 levels and ARAS risk factors (age and systolic pressure). To determine whether these factors had an additive effect on the predictive value of the lncRP11-387H17.6 levels, we performed another ROC curve analysis of lncRP11-387H17.6 and these combined risk factors in the validation group. The diagnostic prediction was increased. The optimal sensitivity of lncRP11-387H17.6 for ARAS increased from 52.5% to 78.9%, although the specificity decreased from 84.8% to 81.9%. The optimal AUC was 0.844 (95% CI = 0.789–0.898) (Figure 4).

## Discussion

The results of this study show that the expression levels of lncRP11-387H17.6 in peripheral blood cells are regulated in ARAS. Older age, systolic blood pressure and lncRP11-387H17.6 were shown to be a significant risk factors for ARAS. A model based on clinical variables may be useful for the clinical identification of high ARAS risk patients who may be proper for renal arteriography at the time of cardiac catheterization.

ARAS results in a progressive loss of renal function and accounts for 90% of cases of renal occlusive vascular disease [2]. Serious ARAS can develop into chronic renal failure within 6 years [23]. Atherosclerosis elicits microvascular and macrovascular dysfunction and tissue structural remodeling, which interact and often exacerbate renal injury [24]. Furthermore, atherosclerosis may trigger defense mechanisms intended to preserve renal structural integrity, thereby facilitating renal scarring [24]. Peripheral blood cells are the most promising compartment for biomarker investigations in the context of ARAS. Compared with invasive renal artery angiography, examination of blood biomarkers is noninvasive and convenient, providing an additional useful component for diagnosing ARAS in high-risk patients.

Recently, an increasing number of lncRNAs have been associated with cardiovascular diseases. For example, the altered expression of lncRNA-P21 has been declared in CAD [25], and hypoxia-inducible factor 1A antisense RNA2 (AHIF) is over expressed in the heart failure [26]. In addition, cyclin-dependent kinase inhibitor 2B antisense RNA1 (ANRIL) may prevent coronary atherosclerosis [27], and increased plasma levels of the lncRNAs H19 and LIPCAR are linked to increased risk of CAD [28]. Recent years, lncRNAs are gaining increasing recognition in chronic kidney disease and renal ischemia–reperfusion injury [29,30], not just in the cardiovascular state. lncRNA XLOC_032768 is beneficial to the anti-apoptosis ability of renal tubular epithelial cells and the regeneration and repair of kidney [29]. lncRNAs may be relevant to osteogenic differentiation, presenting a new perspective into the mechanism of vascular calcification, which is a factor independently associated with cardiovascular death in patients with chronic kidney disease [30]. Due to the above characteristics, lncRNAs are possible candidate biomarkers for the diagnosis of ARAS. The results of this study clearly support the possible hypothesis that lncRNAs are presented in the peripheral blood as candidate biomarkers of ARAS. In the microarray screening and qPCR validation in different groups, we found that RP11-387H17.6 may be a potential novel biomarker with high specificity for the diagnosis of ARAS. After the analysis of the risk factors for ARAS, the high expression of RP11-387H17.6 in blood was found in the elderly and hypertension patients, suggesting the possibility of ARAS, which provided a potential of noninvasive examination for the detection of ARAS.

We used several strategies to decrease the problems associated with statistical analysis of high-throughput biological data (in this case, classifying many genes from a small sample size). First, 43 lncRNAs that were differentially expressed in the peripheral blood cells of individuals with or without ARAS were filtered. To facilitate the confirmation of the target biomarkers, we chose 43 lncRNAs that were upregulated in peripheral blood cells. Only six of these upregulated lncRNAs had average normalized expression intensities >3 with *p*<.05. The expression of these six lncRNAs was verified by qPCR and ROC curve analysis; the AUC of RP11-387H17.6 was 0.826; thus, we further validated this lncRNA. RP11-387H17.6 was selected as the best candidate biomarker for diagnosing ARAS. Among the six lncRNAs with significant expression changes in the renal artery samples, only RP11-387H17.6 could be used as a peripheral blood marker, indicating that the examination of the peripheral blood markers was less sensitive than the examination of the tissue markers. This study shows that expression levels of lncRNAs in blood cells from ARAS patients are extremely variable, and this result is consistent with the fact that only a portion of lncRNAs in tissues can be released to the peripheral blood. As circulating lncRNAs may be correlated with the local lncRNA expression signature in a specific pathology, lncRNAs may be useful potential additional biomarkers.

The stenotic kidney shows significant microvascular rarefaction accompanied by increased fibrosis and a marked deterioration of renal function. The damage and early loss of the renal micro-vessels and the deterioration of the renal angiogenic response (as suggested by the decreased concentrations of vascular endothelial growth factor (VEGF)) are important determinants of the progression of renal injury and likely demarcate the point of often irreversible damage in the stenotic kidney [31]. Our study provides evidence regarding the associations between the peripheral blood cell levels of RP11-387H17.6 and the occurrence of ARAS. Although the functions of the lncRNA RP11-387H17.6 have not been annotated to date, our findings support the hypothesis that this lncRNA may serve as a potential additional indicator of ARAS, and we can infer its possible functions based on the expression of the mRNAs in the same microarray. Notably, the expression of granulocyte-macrophage colony-stimulating factor (G-CSF) was correlated with the expression of the lncRNA RP11-387H17.6. G-CSF is an important survival and proliferation factor for neutrophils and macrophages and can induce the expression of proinflammatory cytokines, thereby enhancing the inflammatory response [32]. Therefore, further studies investigating the relationship between the lncRNA RP11-387H17.6 and G-CSF are warranted. Furthermore, a previous study has demonstrated that tumor cells can recruit monocytes with VEGF and activate monocytes with G-CSF in an NF-kB-dependent manner [33]. We speculate that a connection exists between VEGF and G-CSF in patients with ARAS.

A growing body of evidence from both experimental [34] and human studies [35] clearly indicates the antioxidant effects of statins. All patients in the current study were treated with statins, and most patients received ongoing treatment with ACE inhibitors or ARBs to inhibit the renin angiotensin aldosterone system (RAAS). These two drugs may affect the lncRNA RP11-387H17.6 level in the peripheral blood.

The primary limitation of this study was that the patients were recruited from one hospital in Shenyang, Liaoning; thus, whether our findings also apply to patients in different areas and races is unknown. Therefore, the validity of our findings should be further verified in additional prospective cohorts. Second, kidney tissue was unavailable, and the renal expression of lncRNAs might differ from their expression in peripheral blood cells. Third, due to the lack of data regarding the duration and systemic effects of hypertension, evaluating the possible effects of hypertension on nephroangiosclerosis in ARAS and hypertension patients is challenging. However, based on the clinical profiles of the groups in this study, the ARAS group was likely to have a higher cardiovascular risk than the other groups. Nevertheless, the application of our results to other cohorts should be performed with caution, and further studies are needed. Finally, although this study has shown that lncRNAs had altered in patients with ARAS a potential association with cardiovascular risk factors, LncRNAs have not been studied on the pathological basis of ARAS, only made a hypothesis of some pathological relationship, which needs further experimental verification.

In conclusion, the lncRNA RP11-387H17.6 in peripheral blood cells is differentially expressed between the ARAS patients and the controls. Our findings indicate, for the first time, that the lncRNA RP11-387H17.6 is a potential specificity biomarker of ARAS and may offer an additional component for the study of the disease. Prospective clinical trials should be conducted to determine the usefulness of the lncRNA RP11-387H17.6 as a stable biomarker of ARAS. Furthermore, a better understanding of the role of lncRNA in the post-stenotic kidney may provide novel biomarkers of the outcomes of restorative renal repair in ARAS.

## Supplementary Material

Supplemental Material

## Abbreviations

ARAS: Atherosclerotic renal artery stenosis
lncRNAs: long noncoding RNAs
qPCR: quantitative PCR
ROC: operating characteristic curve
AUC: area under the curve
RAS: Renal artery stenosis
PVD: peripheral vascular disease
CAD: coronary artery disease
OR: odds ratio
TC: total cholesterol
TG: triglyceride
LDL: low-density lipoprotein cholesterol
HDL: high-density lipoprotein cholesterol
eGFR: estimated glomerular filtration rate
NT-ProBNP: N-terminal pro-brain natriuretic peptide
MI: myocardial infarction
HV: healthy volunteers
SBP: systolic blood pressure
CI: confidence interval
