# Supplementary material for: Long noncoding RNA PR11-387H17.6 as a potential novel diagnostic biomarker of atherosclerotic renal artery stenosis
Source: Ren Fail. 2021 Aug 9;43(1):1188–97. doi: 10.1080/0886022X.2021.1956537 (PMC8354168; doi:10.1080/0886022X.2021.1956537)
Supplement: Supplemental Material [file IRNF_A_1956537_SM4694.pdf]

Table S1 Quantitative-PCR primers for the lncRNAs

Gene Name	Forward Primer	Reverse Primer
RP11-387H17.6	CGCAAACAGCAAAAGAACAA	GGCTCTACCAATCAGCAGGA
BC080653	CATCTGCCCTTGGTTGACTT	CCTGGCTACTCCCCTGTTCT
RP1-32B1.4	TAAAGAGGGGTCTGGCTCTG	CCAGCTACTTGACAGGCTGAG
RP5-1068H6.3	CCTGGATCTCTTGACGTGGTT	ATTGGTTGCCAATCTCTTGTCT
GHRLOS	TGATGAATCAGTTGGATGGTG	AGGAGGGCTGAGTGCAAGT
XLOC_009769	CCCAGGAAGAGAGAGGAACC	TGGATAGCAAGTTGGTGCTG
